# Distinct Alpha Connectivity Patterns During Response Inhibition in Alcohol Use Disorder

**DOI:** 10.1002/hbm.70338

**Published:** 2025-09-24

**Authors:** Filippo Ghin, Nasibeh Talebi, Ann‐Kathrin Stock, Christian Beste

**Affiliations:** ^1^ Cognitive Neurophysiology, Department of Child and Adolescent Psychiatry, Faculty of Medicine TU Dresden Dresden Germany

**Keywords:** alcohol use disorder, alpha, effective connectivity, network connectivity, response inhibition, theta

## Abstract

Alcohol use disorder (AUD) is a chronic condition characterized by the inability to control drinking despite experiencing harmful consequences. However, the extent to which excessive alcohol consumption alters the dynamics within the inhibitory control network remains unclear. This study investigates the neurophysiological mechanisms of directed connectivity in alpha and theta frequency bands between cortical regions involved in the interplay between automated and controlled processes during inhibitory control in individuals with mild to moderate AUD. The results indicate that individuals with AUD and healthy controls engage different connectivity networks and direct information transfer mechanisms during response inhibition, especially based on the automaticity of the response contexts. When faced with high demands for inhibitory control, the AUD group exhibits significant differences in directional alpha connectivity in key brain regions associated with the inhibition control network. Additionally, when processing conflicting stimulus information, the AUD group shows enhanced connectivity from the medial prefrontal cortex to early visual processing areas. This highlights an alpha modulatory mechanism that effectively suppresses irrelevant perceptual information. In contrast to alpha, theta seems to play a lesser role in the response inhibition functions of individuals with AUD, as only healthy controls show dynamic neural communication between the prefrontal, temporal, and medial posterior regions. Overall, the study suggests that individuals with AUD engage in a dynamic transfer of information within the alpha frequency band across distinct neural networks depending on the response context during inhibitory control. This might be particularly relevant for understanding the altered inhibitory control associated with this disorder.

## Graphical abstract

Mild to moderate alcohol use disorder (AUD) individuals engage in a dynamic transfer of information within the alpha frequency band across distinct neural networks depending on the response context during inhibitory control.

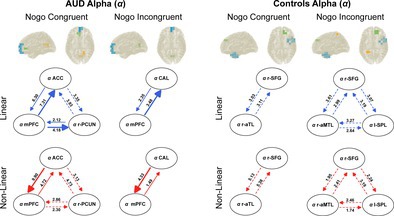

## 1 Introduction

Alcohol use disorder (AUD) is a chronic condition marked by an inability to control or quit drinking despite harmful consequences and is a leading cause of illness and death worldwide (World Health Organization 2018, 2019). Excessive alcohol consumption in individuals with AUD is most likely driven by (automated) alcohol‐related habits and a reduction in inhibitory control functions (Barker and Taylor 2014; Everitt and Robbins 2016; Ghin et al. 2022b; Heinz et al. 2019). Previous research has often overlooked the circumstances in which inhibition demands are affected in AUD, and there is growing interest in a deeper understanding of the cognitive and functional neurobiological mechanisms contributing to the development of AUD (Ghin et al. 2022a, 2022b; Heinz et al. 2019). Dysfunctions in inhibitory control associated with AUD have been linked to changes in cortical and subcortical brain activity (Barker et al. 2015; Everitt and Robbins 2016; Gremel and Costa 2013; Koob and Volkow 2016; Volkow et al. 2012; Volkow and Morales 2015). However, it is still debated whether significant cognitive and neurobiological alterations associated with inhibitory control functions occur already at early stages of the disorder or become evident only in more severe cases (Ghin et al. 2022b; Wilcox et al. 2014). Furthermore, when considering the functional neuroanatomical dimension, evidence suggests that the influence of response context in inhibitory control performance depends on the interregional transfer of information among different frontal, parietal, and temporal areas (Chmielewski et al. 2016; Dippel et al. 2017; Eggert et al. 2023; Ghin et al. 2022a; Ghin et al. 2024; Wendiggensen et al. 2022). In this context, the involvement of different connectivity networks in brain regions associated with inhibition control between individuals with AUD and healthy participants can be anticipated. Importantly, it is relevant to determine whether such differences might occur already in mild to moderate AUD individuals. Additionally, depending on the response contexts in which inhibition is required, distinct connectivity networks are expected to be engaged. Against this background, we set out to determine how inhibitory control functions are affected under automatic versus controlled response contexts to better understand inhibitory control impairments in mild to moderate AUD.

Executing automatic behaviors, which are likely also underlying drinking in AUD, requires minimal cognitive effort. On the other hand, executing controlled actions such as inhibition relies on effortful, top‐down volitional processes. Importantly, controlled and automatic processes are not entirely separate during response inhibition; as they interact and share overlapping neural networks (Chmielewski and Beste 2017; Dippel et al. 2017; Ghin, Stock, and Beste 2022). To understand how inhibitory control is affected in controlled versus automatic response contexts, an experimental paradigm combining a Go/Nogo and Simon task can be used (Ghin et al. 2022a; Ghin, Stock, and Beste 2022; Wendiggensen et al. 2022). In the classic Simon task, the spatial relationship between the task‐irrelevant location of the target stimulus (e.g., left or right) and the position of the response effector (e.g., left or right finger) modulates the level of strength of the prepotent response. The dual‐route model proposes that stimulus–response conflicts emerge from the competition between automatic and controlled response selection processes (De Jong et al. 1994). Controlled processes rely on the identity of the target stimulus (e.g., whether “A” or “B” is presented), which dictates the appropriate response using either the left or right index finger. However, the (task‐irrelevant) target's spatial position automatically triggers a response on the same side. In Go congruent trials, in which stimulus position and response effector coincide, the direct route alone is thus sufficient for correct responses. In incongruent Go trials, where the spatial position requires a response on the opposite side, this automatic response must be inhibited. Thus, conflict arises from the interaction between the direct (automatic) and indirect (controlled) response routes, increasing the possibility of an incorrect response and longer reaction time (i.e., the Simon Effect). Importantly, the effect of stimulus–response congruency is inverted when Nogo trials are presented: performance is worse in congruent Nogo trials than in incongruent Nogo trials. This is because the direct route dominates in Nogo congruent trials, so that response inhibition becomes more complex (leading to more errors than Nogo incongruent trials where the indirect route is already engaged). The interplay of automated and controlled processes in AUD can thus be measured using this experimental approach.

A reliable method to uncover the precise mechanisms of neural processes involved in response inhibition across various brain areas is to investigate the synchronization of neural activity via brain oscillations (Cavanagh and Frank 2014; Cohen 2014; Klimesch et al. 2007; Klimesch 2012), which give rise to the directed transfer of information and coordination across local and distant neural networks important for inhibitory control (Beste et al. 2023; Elmers et al. 2024; Talebi et al. 2024). In particular, low‐frequency oscillations such as theta (*θ*; 4–7 Hz) and the alpha (*α*; 8–12 Hz) band have been associated with inhibitory control functions (Cavanagh and Frank 2014; Klimesch et al. 2007; Klimesch 2012). For instance, there is evidence demonstrating that theta band activity (TBA) is involved in processing conflicting information (Cohen 2014; Folstein and Van Petten 2008; Larson et al. 2014; Pscherer et al. 2019, 2021), including response conflict during inhibitory control (Chmielewski et al. 2016; Dippel et al. 2017). Overall, medial frontal cortex TBA appears to be modulated by the level of top‐down control required for the integration of perceptual information for the purpose of selecting an appropriate (re)action (Cavanagh et al. 2010; Cavanagh and Frank 2014) and has been suggested to enable the coordination of information processing in local and distal neural networks between frontal and parietal cortices (Cavanagh and Frank 2014; Clayton et al. 2015; Huster et al. 2013). It is thus possible that TBA engages different inhibition control networks depending on the prepotent response's automaticity and conflicting information. Therefore, the first goal of this study was to investigate changes in directed information transfer in TBA between cortical regions involved in the interplay of automated and controlled processes during inhibitory control in AUD. However, alpha band activity (ABA) is also important for inhibitory control and the coordination of networked activity in fronto‐parietal cortices (Talebi et al. 2024; Wendiggensen et al. 2023). ABA is central for cognitive functions, such as perception, attention, and working memory, which are critical for inhibitory control (Hanslmayr et al. 2007; Klimesch et al. 2007; Palva and Palva 2007; Scheeringa et al. 2009). A key perspective on ABA's role in inhibition control processes is outlined in the “inhibition timing hypothesis” framework (Klimesch et al. 2007; Klimesch 2012), which suggests that alpha band oscillations might have information‐filtering functions. More precisely, ABA would reflect top‐down inhibitory mechanisms that aid the selection of relevant information by suppressing task‐irrelevant information that might hinder performance (Beste et al. 2023; Prochnow et al. 2022; Talebi et al. 2024). Thus, ABA might be crucial to investigate, but it is currently not well understood to what extent patients in early stages of AUD may exhibit altered alpha connectivity dynamics during response inhibition tasks. Therefore, the second goal of this study was to investigate changes in directed information transfer in ABA between cortical regions involved in the interplay of automated and controlled processes during inhibitory control in mild to moderate AUD.

To examine both goals of this study, it is essential to consider that directed functional connectivity or information transfer between cortical regions can be linear and non‐linear (Babiloni et al. 2005; Ricci et al. 2021; Stam 2005). This is particularly important to consider when it comes to cognitive control functions like response selection and inhibition because top‐down action control integration relies on both feedforward and feedback loops (Ptak 2012; Ptak et al. 2017), where both linear and nonlinear dynamics play a role (Semedo et al. 2019; Shettigar et al. 2022). The importance of distinguishing between linear and non‐linear directed information transfer or connectivity patterns when it comes to inhibitory control processes and the role of TBA and ABA has been corroborated by recent evidence (Talebi et al. 2024). Against this background, this study aimed to investigate linear and non‐linear dynamics in directed connectivity among cortical regions during response inhibition performance in mild to moderate AUD and matched healthy controls in automatic and controlled response contexts. Given the experimental evidence and theoretical frameworks in inhibition control outlined above, we specifically focused on the TBA and ABA activity during response inhibition demands (i.e., Nogo trials). We used an artificial neural network method (nonlinear Causal Relationship Estimation by Artificial Neural Network; nCREANN) (Talebi et al. 2018, 2019) to study directed information transfer in TBA and ABA to provide mechanistic insight into the neurophysiological underpinnings of inhibition control changes in mild to moderate AUD. The nCREANN method was selected due to its capacity to model complex temporal interactions in brain activity, combining interpretability with the flexibility of neural network‐based modeling. While neural networks are often regarded as “black box” models, the analytical formulation of nCREANN allows for explicit interpretation of the input–output mapping and the interactions among time series. This feature makes it particularly suitable for capturing both linear and nonlinear connectivity patterns in brain networks (Elmers et al. 2024; Ghorbani et al. 2024; Talebi et al. 2024).

## 2 Method

### 2.1 Recruitment Procedure and Sample Description

For this study, data from a previous experimental study were used (Ghin et al. 2022a). In the previous study, the eligibility of participants aged 18–40 was determined through a telephone interview, which included a brief version of the SCID structured clinical interview of the DSM‐5 and questions about the applicant's medical history. Applicants were excluded if they reported any withdrawal symptoms that could impair well‐being or performance during a sober test appointment. Additional exclusion criteria included prior medical, neurological, and mental illness records, except for AUD, tobacco use disorder, and acute or lifetime cannabis addiction. Eligible participants did not have any acute or chronic medication intake or other recreational drug addiction that could affect the central nervous system on the testing day. After recruitment, eligible participants were invited to join the study. Each AUD participant was matched with a healthy control participant of the same sex and a similar age (±2 years). At the start of the experimental session, participants read and signed the informed consent form. Breath alcohol concentration (BrAC) was then measured using the “Alcotest 3000” breathalyzer (Drägerwerk, Lübeck, Germany). Participants were required to be sober (BrAC = 0.00%) and provided a urine sample for drug testing (SureStepTM, Innovacon Inc., USA). Positive results for any substances, except for cannabis metabolites (THC–COOH), led to the termination of the study appointment. Participants received EUR 50 as compensation. All applied procedures were approved by the Ethics Committee of the medical faculty of the Technical University of Dresden (EK 513122018) and conducted in accordance with the Declaration of Helsinki. *N* = 148 participants were initially enrolled in the study. From the initial sample, 15 participants were excluded from the analysis for the following reasons: Data from the first two participants were collected and later discarded after minor adjustments to the experimental procedure. A positive urine test for drugs led to the exclusion of three more participants. Data from two more participants were excluded due to technical problems with the experimental equipment. BDI was used to screen for depressive symptoms, and this resulted in the exclusion of three participants who reported moderate depression symptoms (BDI > 19). For two participants, discordant responses at the clinical interview concerning alcohol consumption prohibited the allocation of the participants in the AUD or control group. One participant was excluded due to a prior medical condition not reported in the initial screening. Two participants were excluded due to task performance below chance level (≤ 50% accuracy). Behavioral performance (i.e., accuracy data) was then analyzed separately for each task condition and group to identify potential outliers using the Tukey method in SPSS, resulting in 10 outliers being identified. The final sample comprised *n* = 123 participants, with *n* = 59 (32 males) participants allocated to the AUD group, and the remaining *n* = 64 (28 males) allocated to the control group.

### 2.2 Clinical Assessment

After the initial BrAC and drug assessments, participants completed a series of questionnaires and a structured interview to evaluate their alcohol consumption, depression symptoms, and cognitive abilities. Additional socio‐demographic information and questionnaires (ASSIST and BIS‐15) were collected, but not reported here as they were not considered relevant for the current study. Alcohol abuse and dependence were assessed using the full version of the SCID for DSM‐5. The SCID‐5 for AUD diagnoses AUD according to DSM‐5 criteria for substance use disorder, covering the past 12 months and lifetime AUD based on 11 criteria. Severity is classified as mild (2–3 criteria), moderate (4–5 criteria), or severe (≥ 6 criteria). A past‐year AUD diagnosis is confirmed with at least two criteria met in the past 12 months, while a lifetime AUD diagnosis requires at least two criteria met before the past year. Participants were allocated to the AUD group (≥ 2 AUD criteria) or the control group (≤ 1 AUD criteria) based on their past‐year AUD diagnosis. Other alcohol consumption indices were also evaluated: The AUDs Identification Test (AUDIT) was administered to screen for hazardous and harmful alcohol consumption. Participants reported their daily drinking over the past 3 months to assess drinking frequency and binge‐drinking behavior. Specifically, they were asked, “On how many days did you drink at least one glass of alcohol in the last three months?” (drinking frequency) and “On how many days within the last three months did you drink five or more glasses of alcohol?” (binge drinking). Finally, participants completed the Mini‐Mental State Examination (MMSE) and the Beck Depression Inventory (BDI).

### 2.3 Behavioral Task

**FIGURE 1:**
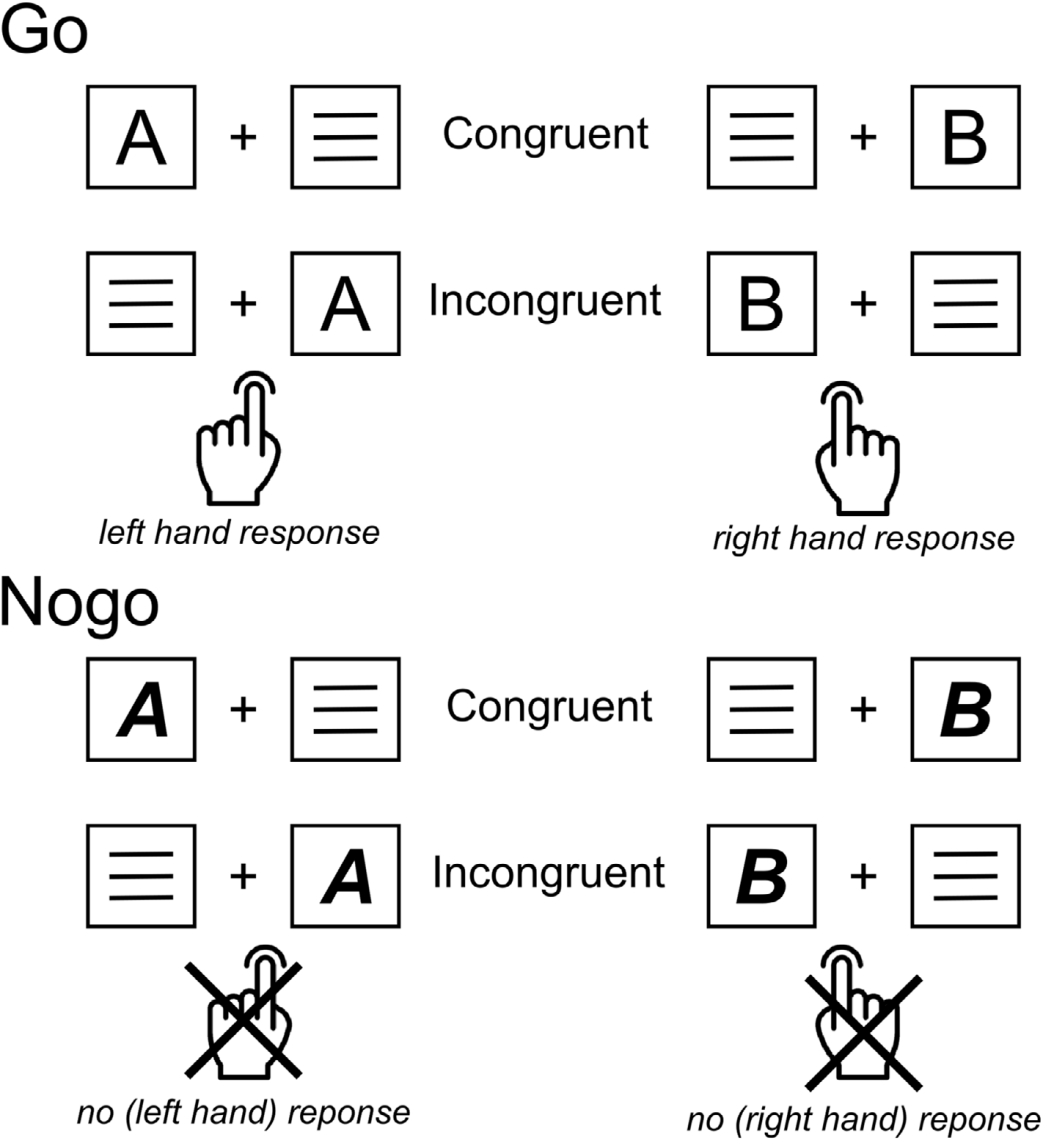
Experimental Simon Nogo paradigm. All possible stimulus–response combinations are illustrated for the Go condition and the Nogo conditions.

Participants were comfortably seated at a distance of 57 cm from a 24″ CRT monitor. A white central fixation cross was continuously displayed in the center of the screen, flanked by two lateral white frame boxes on a black background. This visual setup remained constant throughout the task. During each trial, a single yellow letter (either “A” or “B”) in standard “Arial” font was shown for 200 ms within either the left or right frame box. Simultaneously, a distractor consisting of three horizontal white lines appeared in the opposite box. Participants were instructed to place their left and right index fingers on the left and right Ctrl buttons of a standard German “QWERTZ” keyboard and maintain this position throughout the task. When the letter stimuli appeared in standard font, participants responded as quickly as possible by pressing the corresponding Ctrl button (Go trials). Specifically, they were asked to push the left Ctrl button for the letter “A” and the right Ctrl button for the letter “B,” regardless of the letter's position in the frame boxes. Participants were instructed to inhibit any response if the letters were displayed in a combined bold–italic font (Nogo trials). Trials where the letter stimulus matched the spatial position of the response hand were coded as congruent (e.g., “A” on the left, “B” on the right). Trials where the letter was in the opposite position were coded as incongruent (e.g., “A” on the right, “B” on the left). The experimental paradigm consisted of four primary conditions: (1) congruent Go trials, (2) incongruent Go trials, (3) congruent Nogo trials, and (4) incongruent Nogo trials. Participants completed 720 trials, with 70% (504 trials) being Go trials and 30% (216 trials) being Nogo trials (with 50% of each of those trials bring congruent and the other 50% incongruent). The inter‐trial interval (ITI) varied between 1300 and 1700 ms. Go trials were coded as “hits” if a correct response was made within 0–1700 ms after stimulus onset, and as “errors” if an incorrect response was made within this window. If no response was registered within 1700 ms, the Go trial was coded as a “miss.” Nogo trials were coded as correct omissions if no response was made within 1700 ms, and as “false alarms” if any response was made within this period. The ITI began either when a response was made or after 1700 ms without a response. The experiment was divided into six blocks of 120 trials each, with an equal distribution of trial types and conditions in each block. Participants could take self‐timed breaks between blocks and resume the task with a button press. The entire experiment took approximately 30 min to complete. The experiment was programmed and behavioral data were recorded using Presentation software (Neurobehavioral Systems Inc., Berkeley, California, United States), and is illustrated in Figure 1. After the Simon Nogo task, participants engaged in two additional but unrelated experimental paradigms.

### 2.4 Behavioral Analysis

The behavioral performance data was analyzed with a repeated‐measures ANOVA accounting for congruency (congruent vs. incongruent) and trial type (Go vs. Nogo) as within‐subject factors and group (AUD vs. controls) as a between‐subject factor. Since the pattern of results obtained via this analysis is analogous to previously reported findings (Ghin et al. 2022a), the details can be found in the Supporting Information.

### 2.5 EEG Recording and Analysis

The EEG signal was recorded using 60 evenly spaced (equidistant) Ag–AgCl electrodes. A BrainAmp amplifier (Brain Products GmbH, Gilching, Germany) was used for recording with a sampling rate of 500 Hz. The reference electrode was placed at Fpz (*θ* = 90, *φ* = 90), while the ground electrode was positioned at coordinates *θ* = 58, *φ* = 78. Offline, the data were down‐sampled to 256 Hz using Brain Visual Analyzer software (Brain Products GmbH). Furthermore, a band‐pass filter (IIR) with a range of 0.5–40 Hz and a slope of 48 dB/octave was applied for post‐processing, along with a 50 Hz notch filter. Noisy or flat‐line channels were discarded, and the recording was re‐referenced against the computed average signal of the remaining electrodes. Manual inspection was applied to the raw data in order to remove non‐periodic technical artifacts. Periodic artifacts, such as eye blinks, saccades, and cardiovascular activity, were corrected through independent component analysis using the Infomax algorithm. A second manual inspection was then carried out to remove any remaining artifacts. Discarded channels were reconstructed through topographic interpolation. Only data from trials with correct behavioral responses (Nogo correct omissions) were included in the analysis. The data were segmented into the two Nogo conditions, each starting at −2000 ms and ending at 2000 ms relative to stimulus onset. An automatic artifact rejection procedure was used to remove segments showing a maximum value difference greater than 200 μV within a 200 ms window, or a minimum of less than 0.5 μV within a 100 ms window. Additionally, the EEG signal time series in the theta (4–7 Hz) and alpha (8–12 Hz) frequency bands were extracted using Hamming‐windowed sinc FIR filters. These filtered signals were subsequently used for beamforming and connectivity analysis.

#### 2.5.1 Beamforming Analysis

We reconstructed sources from EEG signals to estimate the brain's activity at specific anatomical locations, reducing interference from scalp signals. By combining source reconstruction techniques with connectivity analysis, we reduced volume conduction effects and provided an objective means to identify brain regions involved, avoiding reliance on predefined cortical segmentation (Sohrabpour et al. 2016). The sources were reconstructed using the linearly constrained minimum variance (LCMV) beamforming technique using FieldTrip (Van Veen et al. 1997). Initially, a typical spatial filter was calculated from the time‐locked average of trials, with both Nogo congruent and Nogo incongruent conditions concatenated. Next, condition‐specific spatial filters were generated using the previously computed standard filter. Finally, the time courses of source signals for each trial were reconstructed using the condition‐specific spatial filters. The clusters of the voxels in theta and alpha activity in the LCMV‐beamformed data were then defined by applying the Density‐Based Spatial Clustering of Applications with Noise (DBSCAN) algorithm (Ester et al. 1996) onto the Neural Activity Index (NAI) of the sources. The voxel selection was limited to functional neuroanatomical regions with high activity, as the threshold was set to the top 2% of the NAI distribution within labeled regions in the Automatic Anatomical Labeling atlas (Tzourio‐Mazoyer et al. 2002). The minimum cluster size was set to two voxels, with an epsilon value of 1.5 times the voxel edge length to identify neighboring voxels. Clusters were visually inspected for further analysis based on their voxel count and anatomical labels. For each selected cluster, the average time course of all voxels within the cluster was used as the representative activity estimate in the connectivity analysis.

#### 2.5.2 Directed Functional Connectivity

To evaluate the directed connectivity patterns among the clusters established by the DBSCAN algorithm, we used the nCREANN method (Talebi et al. 2019). nCREANN uses an artificial neural network (ANN) to estimate directed connectivity based on a nonlinear Multivariate Autoregressive (nMVAR) model. In an nMVAR model, the current samples of brain regions are generated based on interactions between previous areas. Typically, the nMVAR model is used to represent temporal causality, in which the cause impacts the future. The nCREANN approach captures linear and nonlinear dynamics of the information flow among cortical areas (in contrast to standard linear methods focusing only on linear MVAR models). Due to the complex and nonlinear behaviors of the nervous system ranging from a single neuron to the system level (He and Yang 2021; Kodama and Galán 2019; Yang et al. 2018), linear methods carry the risk of oversimplifying the intricate dynamics of brain function. Nonlinear interactions, in particular, play a crucial role in organizing information flow between cortical regions (Kodama and Galán 2019; Yang et al. 2018), and studies have demonstrated the significance of both linear and nonlinear mechanisms for advancing our understanding of neurodynamics on a macro scale (Chen et al. 2009; Cifre et al. 2021; Ferdousi et al. 2020; Friston 2001; Nozari et al. 2020).

Within the nMVAR model, the current sample of each signal is expressed as a (non)linear function of its past values and past values of other signals, enables an inference of temporal causality where a cause affects the future. For a given time series $x\left(n\right)\in {\mathbb{R}}^{M}$ of length *L*, an nMVAR model of order p is defined as

(1) $$x\left(n\right)=f\left(x_{p}\right)+\sigma \left(n\right)$$

where $x_{p}={\left[x_{1}\left(n-1\right),x_{2}\left(n-1\right),\cdots ,x_{M}\left(n-p\right)\right]}^{T}$ is the vector of p past samples of (*M*) multivariate time series. The noise vector, $\sigma \left(n\right)={\left[\sigma_{1},\sigma_{2},\ldots ,\sigma_{M}\;\right]}^{T},$ is the model residual, and the nonlinear function $f\left(.\right)$ quantitatively describes how the p previous samples cause the future values. In the nCREANN method, the functions f is divided into linear and nonlinear part:

(2) $$f=f^{\mathrm{Lin}}+f^{\text{NonLin}}$$

Linear Connectivity, ${\mathrm{LC}}_{i\to j}$, is calculated using the $f^{\mathrm{Lin}}$, as the linear impact of *i*th cluster on the *j*th cluster, and the Nonlinear Connectivity, ${\mathrm{NC}}_{i\to j}$, is derived from the information embedded in the $f^{\text{NonLin}}$, allowing the inferring the nonlinear causal effect of $x_{i}$ on $x_{j}$.

In the present study, the nCREANN was applied to the time courses of the LCMV‐derived sources in Nogo congruent and Nogo incongruent conditions for “AUD” and “Control group” groups in different frequency bands. The data points of the trials in the time interval from 0 to 1000 ms after stimulus onset were considered for the connectivity analysis. All trials were concatenated to create a data set with a sufficient length for training the network. Akaike and Schwartz criteria (Neumaier and Schneider 2001) were used to assess the best model order (*p* = 10). p was considered the same for all conditions. A Multilayer Perceptron neural network with one hidden layer and 10 hidden neurons was trained. The network's input was the $x_{p}$ and the it tries to predict $x\left(n\right)$ as its output. The training algorithm was gradient descent error back‐propagation with momentum (*α*) and adaptive learning rate (*η*). For sake of generalization, the early stopping technique was applied. The 10‐fold permuted cross‐validation technique was applied, and in each fold, the data was divided into 80% training, 10% validation, and 10% testing sets. The network parameters were updated in the “incremental” mode (each time an input is presented to the network), with random initial parameters in the range of (−0.5, 0.5). The model's validity was evaluated using Mean Square Error (MSE) and *R* ^2^ values for the training and test data. A low MSE in both datasets indicates the model's fitting and generalization capability. Additionally, *R* ^2^ values approaching 1 further highlight the model's accuracy in capturing the underlying patterns and its goodness of fit. Additionally, a randomization test with the creation of 100 data sets based on the time‐shifted surrogate technique was used to evaluate the significance of the connectivity values (Papana et al. 2013). This method eliminates any causal relationship between the signals without altering the dynamics of each time series. When applying nCREANN to the surrogate data, the network configurations were precisely the same as those for the original data. The connectivity patterns were displayed on a schematic plot, with the arrows showing the information flow from one cluster (circle) to another. The coordinates of the source clusters were achieved with the DBSCAN analysis (Section 2.5.1). The thickness of the arrows and the arrowhead size were proportional to the connectivity values. The non‐self‐connections were plotted for the average values across all subjects.

## 3 Results

### 3.1 Alcohol Consumption and Sociodemographic Data

Alcohol consumption indices and sociodemographic data for both the AUD and the control group are reported in Tables 1 and 2, respectively. Mann–Whitney *U*‐tests were used to test for differences between the AUD and control groups. Predictably, significant group differences were found for all alcohol consumption indices (all *p* > 0.05). Regarding the sociodemographic data, no difference in age and MMSE score was found between AUD and the control group. However, a significant difference in education and BDI score was found. Specifically, the AUD group reported higher education and BDI scores than the control group. It is worth noting that the difference in the years of education is, however, less than a year, and the BDI scores for both groups revealed only minimal depression symptoms. To ensure that both education and BDI score had no influence on accuracy, Pearson's correlations were performed between BDI scores, years of education, and performance in the task. Results showed no significant correlation in all the behavioral measures (all *p* > 0.05). Detailed results are reported in Table S1. Additionally, detailed information on the conversion into standard drinks and more detailed data for 1‐year and lifetime AUD criteria are also reported in Tables S2 and S3.

**TABLE 1:** Sociodemographic measures for AUD and control participants.

	AUD		Controls		*z*	*p*
	*M* (SEM)	Range	*M* (SEM)	Range		
Age (years)	27.07 (0.8)	18–40	27.06 (0.7)	18–40	−0.257	0.798
Years of education	12.70 (0.1)	9–13	12.16 (0.2)	9–13	−2.595	0.009*
BDI	6.59 (0.7)	0–19	4.38 (0.5)	0–17	−2.216	0.027*
MMSE	29.14 (0.1)	26–30	29.32 (0.1)	27–30	−1.258	0.208

*Note:* * indicates significant results.

**TABLE 2:** Alcohol consumption measures for AUD and control participants.

	AUD		Controls		*z*	*p*
	*M* (SEM)	Range	*M* (SEM)	Range		
1‐year AUD criteria	4.41 (0.22)	2–9	0.25 (0.05)	0–1	−9.894	< 0.001*
Lifetime AUD criteria	4.17 (0.34)	0–10	0.66 (0.12)	0–4	−7.892	< 0.001*
AUDIT score	14.81 (0.70)	5–31	5.47 (0.39)	0–16	−8.608	< 0.001*
Drinking frequency	41.14 (2.52)	6–84	18.16 (1.85)	0–60	−6.270	< 0.001*
Binge drinking frequency	16.86 (1.89)	0–66	3.10 (0.57)	0–24	−6.734	< 0.001*

*Note:* Values are reported as the mean and standard error of the mean. The number of drinks was extrapolated from the amount and type consumed alcoholic beverages. (Binge) drinking frequency is defined as the number of (binge) drinking events in the 3 months prior to the study. * indicates significant results.

### 3.2 Behavioral Results

Given that the focus of the current study was to investigate the functional role of linear and non‐linear connectivity during inhibitory control function in AUD compared to healthy controls, a report of the main behavioral results of the same data set published in Ghin et al. (2022a) is provided in the Supporting Information. Contrary to the original hypothesis of impaired inhibition in AUD, behavioral results actually showed an overall better inhibition response in the AUD group, as compared to the control group. Both the AUD and the control group showed the typical Simon effects, with better performance for Go congruent compared to Go incongruent trials and better performance for Nogo incongruent compared to Nogo congruent trials. Furthermore, the AUD group showed a smaller Simon Nogo effect (i.e., Nogo congruent *minus* Nogo incongruent trials) than the control group. The smaller Simon effect in the AUD group might suggest that, compared to healthy controls, AUD participants were less affected by the level of automaticity of the prepotent response, resulting in a better overall inhibition performance. Furthermore, an additional ANCOVA analysis showed that AUD maintained an overall better inhibition performance even when controlling for the number of AUD lifetime criteria (see Supporting Information). In Ghin et al. (2022a) we also investigated whether alcohol‐related factors influenced the effect of automaticity on response inhibition. Specifically, it was found that higher drinking frequency, instead of the number of AUD criteria, was associated with the reduced modulation of the response inhibition performance. For this reason, we limited the neurophysiological analyses to the NOGO condition (see below). For a complete account of the behavioral results, we refer the reader to Ghin et al. (2022a).

### 3.3 Neurophysiological Results

**FIGURE 2:**
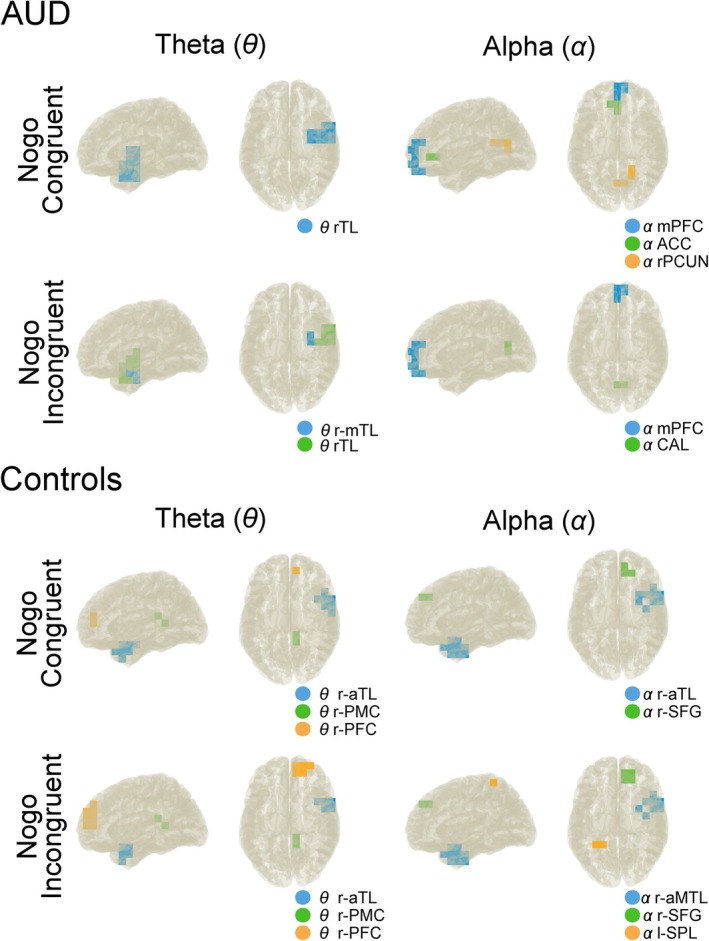
Clusters with the 2% most active voxels in theta and alpha frequency bands for Nogo congruent and Nogo incongruent conditions in the AUD and control group. Brain regions in theta frequency band: mPFC, medial prefrontal cortex; r‐aTL, right anterior temporal lobe; R‐mTL, right medial temporal lobe; r‐PFC, right prefrontal cortex; r‐PMC, right posterior medial cortex; r‐TL, temporal lobe. Brain regions in alpha frequency band: ACC, anterior cingulate cortex; CAL, calcarine cortex; l‐SPL, left superior parietal lobe; MPFC, medial prefrontal cortex; r‐PCUN, right precuneus; r‐SFG, right superior frontal cortex.

The Beamforming/DBSCAN‐derived clusters were determined separately for both conditions and both groups. The regions with the highest activity (exceeding the 90% of the surrogate data) were plotted on a template head model derived from a “colin27” brain (in MNI152 space) for Nogo congruent and Nogo incongruent conditions at theta and alpha frequency bands. Results are shown in Figure 2, where clusters are shown in different colors. The cluster details are provided in Table 3.

**TABLE 3:** Cluster labels and corresponding Brodmann areas for the three frequency bands.

	*α* Theta^a^		*α* Alpha^b^	
	Label	Brodmann	Label	Brodmann
AUD				
Nogo	*θ* r‐TL	38, 21, 43	*α* mPFC	9, 10, 11
Congruent			*α* ACC	32
			*α* r‐PCUN	18, 19
Nogo	*θ* r‐mTL	28, 34	*α* mPFC	9, 10, 11
Incongruent	*θ* r‐TL	38, 21, 43	*α* CAL	17
Controls				
Nogo	*θ* r‐aTL	21, 37, 38	*α* r‐aTL	21, 37, 38
Congruent	*θ* r‐PMC	23, 31	*α* r‐SFG	9, 10
	*θ* mPFC	10		
Nogo	*θ* r‐aTL	21, 37, 38	*α* r‐aMTL	21, 37, 38
Incongruent	*θ* r‐PMC	23, 31	*α* r‐SFG	9, 10
	*θ* r‐PFC	9, 10	*α* l‐SPL	7

Abbreviations: ACC, anterior cingulate cortex; CAL, calcarine cortex; l‐SPL, left superior parietal lobe; mPFC, medial prefrontal cortex; r‐aTL, right anterior temporal lobe; r‐mTL, right medial temporal lobe; r‐PCUN, right precuneus; r‐PFC, right prefrontal cortex; r‐PMC, right posterior medial cortex; r‐SFG, right superior frontal cortex; r‐TL, temporal lobe.

^a^ *θ*: theta frequency band.

^b^ *α*: alpha frequency band.

With regard to theta frequency in the AUD group in Nogo congruent trials, there was only one cluster of voxels. Therefore, this frequency band was excluded for this group, and the nCREANN was just applied for the alpha band. For the control group, both theta and alpha frequency bands were analyzed for the connectivity patterns.

**FIGURE 3:**
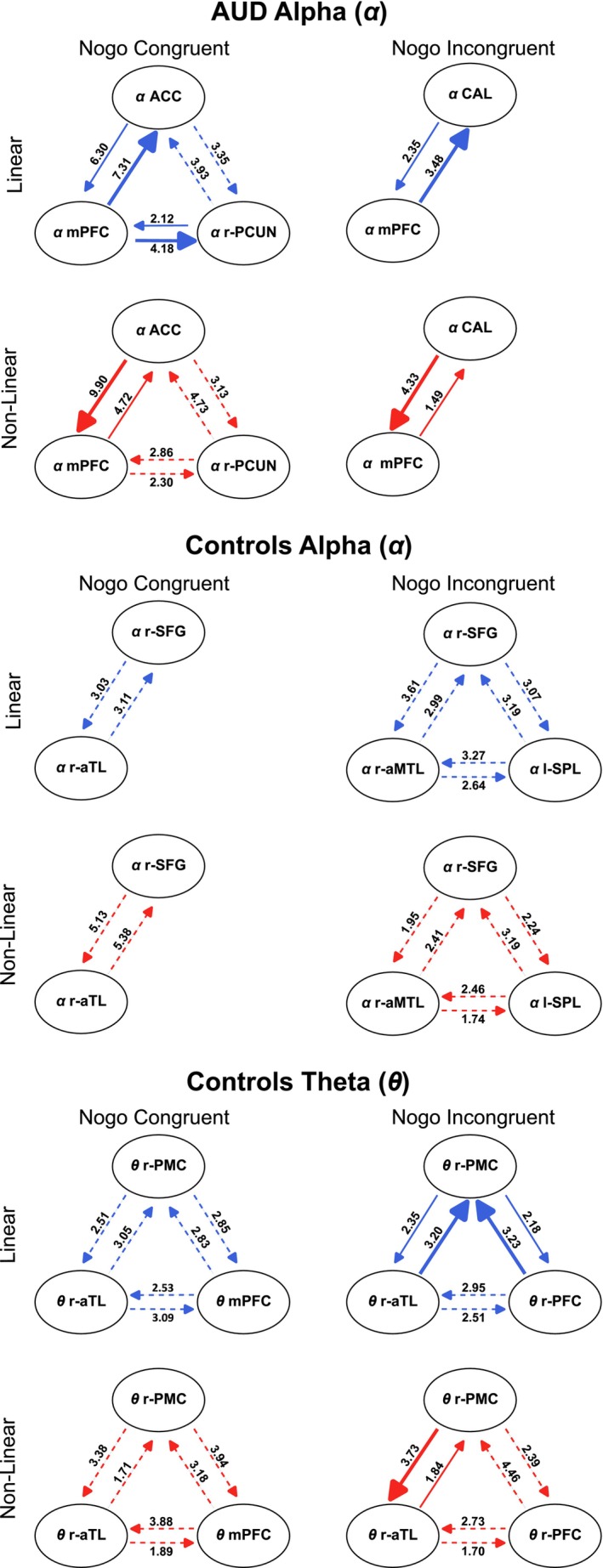
Linear (top) and nonlinear (bottom) directed connectivity pattern in Nogo congruent and Nogo incongruent conditions for theta and alpha frequency bands in the AUD and control group. Please note that no connectivity pattern was found for the theta band in the AUD group. Brain regions in theta frequency band: mPFC, medial prefrontal cortex; r‐aTL, right anterior temporal lobe; R‐mTL, right medial temporal lobe; r‐PFC, right prefrontal cortex; r‐PMC, right posterior medial cortex; r‐TL, temporal lobe. Brain regions in alpha frequency band: ACC, anterior cingulate cortex; CAL, calcarine cortex; l‐SPL, left superior parietal lobe; MPFC, medial prefrontal cortex; r‐PCUN, right precuneus; r‐SFG, right superior frontal cortex.

To evaluate the brain network organization in each frequency band, nCREANN was applied to the time courses of the source signals in theta (control group) and alpha frequency bands (control group and AUD). The model validation measures for all groups/condition were ${\mathrm{MSE}}_{\text{train}}\leq 0.030\pm 0.010$, ${\mathrm{MSE}}_{\text{test}}\leq 0.023\pm 0.015$, and $R_{\text{train}}^{2}\geq 0.961\pm 0.121$ and $R_{\text{test}}^{2}\geq 0.949\pm 0.250$. Figure 3 shows the obtained average linear (top‐blue) and nonlinear (bottom‐red) connectivity schematic patterns. The average connectivity values across all subjects are shown in the arrows. They have been multiplied by 100 to improve the visualization of the figures, making the numbers easier to read and interpret. The connectivity arrows were plotted in solid lines if there was a statistically significant difference (*p* < 0.05) between the two directions of the information flow. Thick solid arrows and arrowheads indicate significant differences in the connectivity strength. Dashed lines were used in case of non‐significant differences (*p* > 0.05). As shown in Figure 3 (top section), there is significant linear connectivity in the AUD group in the alpha frequency band from *α* medial prefrontal cortex (mPFC) to the *α* calcarine sulcus (CAL) (3.48) for Nogo incongruent trials, and from *α* mPFC to *α* anterior cingulate cortex (ACC) (7.31) and r‐PCUN (4.18) for Nogo congruent trials, which are significantly higher than the opposite directions. The adverse pattern is seen for the nonlinear connectivity (*α* CAL to *α* mPFC is 4.33, and *α* ACC to *α* mPFC is 9.90) in this figure. Furthermore, most connections did not show a significant difference in their direction in the control group (Figure 3, middle and bottom section). In this group, only the linear connectivity from *θ* r‐aTL (3.20) and *θ* rPFC (3.23) to *θ* r‐posterior middle cortex (PMC) is significantly higher than in the other direction. The inverse pattern is seen for the nonlinear connectivity from *θ* r‐PMC to *θ* r‐aTL (3.73) in the Nogo incongruent condition for the control group.

Furthermore, the statistical analysis of the average connectivity (i.e., mean of all connections), representing the network's strength, revealed a significant difference between congruent and incongruent Nogo trials for the AUD group within the alpha frequency band (*p*_Linear connectivity_ = 6.93e^−08^ and *p*_Nonlinear connectivity_ = 1.414e^−3^). However, no significant condition difference was found in the theta and alpha frequency bands for the control group. Within the alpha frequency band, there was a significant group difference in the Nogo congruent condition (*p*_Linear connectivity_ = 1.975e^−07^ and *p*_Nonlinear connectivity_ = 3.414e^−04^). In the Nogo incongruent condition, a significant difference between the groups was observed only in nonlinear connectivity (*p*_Nonlinear connectivity_ = 0.021).

## 4 Discussion

The neurophysiological underpinning of cognitive control changes in AUD remains poorly understood. Most importantly, little is known about the consequences of alcohol abuse on theta and alpha band oscillations and their dynamic information transfer across brain areas—despite their critical role in cognitive control functions. Against this background, our study investigated linear and non‐linear directed connectivity alpha and theta across spatially distinct cortical regions during response inhibition performance in AUD and healthy controls. Contrary to our initial hypothesis, we found that individuals with mild to moderate AUD had a relatively small, but significantly better inhibition performance compared to healthy controls. Furthermore, the AUD group showed a smaller Simon Nogo effect (Nogo congruent *minus* Nogo incongruent trials). Thus, the AUD group showed a smaller modulation of inhibition performance by the level of automaticity versus control exerted for the prepotent Go response. These findings were unexpected, and several factors may account for them. Although inhibitory deficits are typically associated with problematic drinking and relapse in AUD, there is still an ongoing debate about whether these impairments occur early in the disorder or only in later and more severe stages. Variability in the findings might arise from differences in drinking severity and task design across studies (Ghin et al. 2022b). For example, the salience of the stimuli might be a critical factor: When alcohol‐related stimuli are used, inhibition control deficits regularly become evident in AUD compared to healthy controls (Corbit et al. 2012; Garbusow et al. 2014; Sommer et al. 2017). However, individuals with AUD and healthy controls might show comparable inhibition performances when neutral stimuli are used (Kamarajan et al. 2005). These findings indicate a complex relationship between AUD severity and impairments in inhibitory control, with the latter particularly influenced by the response context and the subjective salience of the stimuli triggering the prepotent response. Yet, the focus of this study was how directed information transfer between cortical regions associated with ABA and TBA is (differently) modulated in AUD. We had hypothesized that individuals with AUD might entertain different alpha and theta connectivity networks compared to healthy controls, and that the dynamics of the neural architecture could vary depending on the automaticity of the response contexts during response inhibition. The obtained findings support these hypotheses. Below, we discuss findings for alpha and theta frequency oscillations in separate sections in detail.

### 4.1 Directed Connectivity in ABA

In the ABA network, the AUD group showed differential directed linear and non‐linear connectivity strength between the mPFC and the ACC in the most challenging (Nogo congruent) condition. Higher ABA has been associated with inhibitory modulation of sensory information (Klimesch et al. 2007; Klimesch 2012) which is particularly important if there is information that should not be processed. This inhibitory state is likely governed by top‐down attentional mechanisms (Klimesch et al. 2007; Klimesch 2012) and other studies also support that such higher inhibitory controlled gating of information is required in Nogo congruent trials (Chmielewski and Beste 2017; Ghin, Stock, and Beste 2022). From a neuroanatomical perspective, the mPFC and the ACC have been linked to cognitive control in managing cognitive load (Botvinick et al. 2004; Botvinick 2007; Botvinick and Cohen 2014; Paus et al. 1993; Ridderinkhof et al. 2004; Rubia et al. 2001). Furthermore, the AUD group presented a significant difference in linear connectivity strength between the mPFC and the precuneus in Nogo congruent trials. Although the precuneus' role in cognitive control remains somewhat debated, evidence suggests that increased activation of the precuneus corresponds to greater top‐down allocation of cognitive resources (Cavanna and Trimble 2006; Ghin et al. 2022a; Koyun et al. 2023; Stock et al. 2016). This reflects the function of ABA in facilitating communication between brain regions, illustrating the interaction between top‐down and bottom‐up processes that occur between frontal and occipital‐parietal areas. Moreover, connectivity was identified between the mPFC and the CAL, a portion of the primary visual area in incongruent Nogo trials. Such connection suggests the presence of direct transfer of information between the mPFC and the primary visual cortex. This suggests an interaction between top‐down control and visual bottom‐up processes. In this regard, previous research demonstrated that the visual information processed in the primary visual areas can be modulated by attention and cognitive demands (Gilbert and Li 2013; Kamiyama et al. 2016; Muckli 2010). The interplay of alpha connectivity patterns between frontal and occipital regions in Nogo incongruent trials evident in the AUD group thus suggests the presence of an alpha‐based mechanism for the inhibition of irrelevant perceptual information.

Unlike the AUD group, the control group did not show differential strength in the linear and non‐linear connectivity directions between the identified cortical areas in the alpha band. They further presented a different neural architecture in both Nogo congruent and incongruent trials: The results showed both linear and non‐linear connectivity between the temporal and medial‐temporal lobe (MTL), the superior frontal gyrus (SFG), and the superior parietal lobe (SPL). Importantly, the connectivity architecture involving the SPL was present only in Nogo incongruent trials. The observed results indicate a bi‐directional transfer of information in the neural network between cognitive control‐related areas and temporal and superior parietal areas known as part of the ventral and dorsal visual stream, respectively (Goodale and Milner 1992). The ventral stream is critical for recognizing and processing visual features important for planning an appropriate course of action (Eggert et al. 2023; Grill‐Spector and Weiner 2014). On the other hand, the dorsal stream has been associated with the programming and online control of fine movements based on bottom‐up visual information (Gallivan and Goodale 2018; Milner and Goodale 2008). Notably, the role of the SPL emerges specifically when an incongruency between stimulus location and prepotent go response occurs (i.e., Nogo incongruent trials). A critical information transfer between frontal inhibitory top‐down inputs and bottom‐up information about the location of the visual stimulus from higher levels of the visual streams can explain why the discrepancy in the stimulus–response spatial relationship reduces the level of automatic prepotent response. It is also important to note that connections between temporal and superior parietal regions about stimulus information align with current evidence suggesting that (unlike previously thought) the ventral and dorsal visual streams should not be considered entirely functionally distinct networks (Goodale and Milner 1992). Instead, there is a strong cross‐communication between these areas, indicating a dynamic and flexible interaction among different visual processing stages and their role in perception‐action integration (Cloutman 2013; de Haan et al. 2018).

Compared to the control group, AUD participants displayed differential strength in linear and non‐linear alpha connectivity dynamics in both Nogo congruent and Nogo incongruent trials. The greater specialization of the information transfer in distinct operating inhibition networks, depending on the automaticity of the response context, suggests a more efficient engagement of inhibition demands, which ultimately seems to translate into better behavioral performance of individuals with mild to moderate AUD.

### 4.2 Directed Connectivity in TBA

Concerning TBA, source reconstruction results for the AUD group only showed a single activity cluster in the temporal lobe for Nogo congruent trials, while temporal and medial‐temporal lobe clusters were obtained for the Nogo incongruent trials. Given that at least two spatially distinct areas are necessary to perform nCREANN, connectivity analysis was conducted exclusively for the control group. The theta band network for the control group showed bi‐directional connectivity for linear and non‐linear connectivity among spatially distinct brain regions. Both congruent and incongruent Nogo trials engaged a theta connectivity network involving the PMC, the TL, and the prefrontal regions. Importantly, the PMC comprises the posterior cingulate cortex, a hub for distinct distributed brain networks that supervise the allocation of attentional demands (Leech et al. 2011; Leech and Sharp 2014). Yet, a significant directional difference in the connectivity strength was only evident for Nogo incongruent trials. It has been conceptualized that TBA could act as an “alert signal” in inhibition control, guiding ongoing goal‐directed actions, shifting behavioral strategies, and increasing cognitive control (Cavanagh and Frank 2014). Thus, TBA might be particularly relevant when conflicting stimulus information needs to be resolved. Therefore, a distributed theta network controlling the transfer of information between the PCM, the PFC, and the TL in Nogo incongruent trials might explain the typically better performance in that condition of the Simon Nogo task.

### 4.3 Average Linear and Non‐Linear Connectivity

Finally, statistical analysis of average connectivity, representing overall network strength, showed a significant difference between congruent and incongruent Nogo conditions within the alpha frequency band in the AUD group. However, no significant differences emerged between these conditions for the control group in either the alpha or theta bands. Additionally, a significant difference between the AUD and control groups was observed in the alpha band for the Nogo congruent condition. For the Nogo incongruent condition, a significant difference between the groups was found only in nonlinear connectivity. The results indicate that, in comparison to control participants, the AUD group demonstrates a better ability to differentiate between high and low demands during inhibitory control tasks. Specifically, the overall increased connectivity strength in cognitive control‐related areas, such as the mPFC and ACC, during Nogo congruent trials might reflect a higher level of engagement and more efficient inhibitory control processes.

### 4.4 Limitations

Despite these findings, our method has some limitations. In our study, we recruited participants with AUD from the general population who had not previously received a diagnosis and only aimed at investigating participants that reported mild to moderate AUD. However, the duration of alcohol abuse is a critical component of the disorder, and it likely relates to both behavioral and neurophysiological factors. In our study, participant group selection was determined using the SCID structured clinical interview of the DSM‐5, which focuses on different aspects related to drinking abuse to assess the severity of the disorder. However, this structured interview does not specifically address the duration of the disorder. Understanding the duration or chronicity of alcohol abuse can be particularly complex, especially in cases where no formal diagnosis has been made before. This is especially relevant for individuals with mild to moderate AUD, who may not yet fully recognize their drinking habits as problematic. Another limitation concerns the nCREANN analysis. Like other machine learning approaches, nCREANN requires a sufficient amount of data for effective training. To address this, we utilized single‐trial EEG signals to increase the effective data size. Moreover, we applied nCREANN to the full segment of the task‐related signal to estimate an overall connectivity profile in this study. We acknowledge that incorporating a time‐varying implementation of nCREANN could offer more detailed insights into the dynamic evolution of brain connectivity.

## 5 Conclusions

In summary, our results show that individuals with AUD and healthy controls engage in different connectivity networks‐directed information transfer during response inhibition. Distinct linear and non‐linear connectivity patterns emerge based on the automaticity of the response contexts. Notably, when faced with high demands for inhibitory control, the AUD group exhibited significant directional alpha connectivity differences in key brain regions resembling the inhibition control network. When processing conflicting stimulus information, the AUD group additionally showed enhanced connectivity from the mPFC to early visual areas, highlighting an alpha modulatory mechanism that effectively suppresses irrelevant perceptual information. Contrary to ABA, TBA appears less involved in AUD response inhibition functions, with only healthy controls showing dynamic neural communications between the prefrontal, temporal, and medial posterior regions in that frequency band. The dynamic transfer of information within the alpha frequency band across neuroanatomically distinct neural networks in AUD might be particularly relevant to better frame altered inhibitory control in mild to moderate AUD.

## Author Contributions

**Filippo Ghin:** conceptualization, software, investigation, formal analysis, writing – original draft, visualization, funding acquisition. **Nasibeh Talebi:** software, formal analysis, writing – original draft, visualization. **Ann‐Kathrin Stock:** conceptualization, software, writing – reviewing and editing, supervision, funding acquisition. **Christian Beste:** conceptualization, writing – original draft, writing – reviewing and editing, supervision, funding acquisition. All authors had full access to the data, gave final approval for publication, and agreed to be held accountable for the work performed therein.

## Ethics Statement

All participants provided written informed consent and received a financial reimbursement for their participation in the study. The ethics committee of the TU Dresden approved this study.

## Consent

The authors have nothing to report.

## Conflicts of Interest

The authors declare no conflicts of interest.

## Supporting information


**Table S1:** Pearson's correlations.
**Table S2:** Number of standard drinks per serving size of different alcoholic beverages in liters (l).
**Table S3:** Frequency table for AUD 1‐year criteria and AUD lifetime criteria for AUD and control participants.
**Figure S1:** Behavioral results. The box plots illustrate the mean accuracy in percent (%) for the four experimental conditions. Accuracy in the Go condition was measured in correct responses. Accuracy for the Nogo condition was a measure of correct omission. Asterisks (*) indicate significant differences at *p* < 0.05. Error bars represent the 95% confidence interval. The performance of participants with alcohol use disorder (AUD) is presented in blue, while that of healthy controls is presented in green.
**Figure S2:** Time‐frequency representation averaged over all channels for AUD (top panels) and control group (bottom panels) for Nogo congruent and Nogo incongruent trials in the time frame used for the nCREANN analysis (1 s after stimulus onset). No significant difference was found between Nogo congruent and Nogo incongruent in alpha and theta band power for the AUD (all *t* < 1.029, all *p* > 0.308) and the control groups (all *t* < 1.542, all *p* > 0.128). No significant difference was also present between the AUD and the Control group in alpha and theta frequency for Nogo congruent trials (All *t* < −1.420, all *p* > 0.79) and Nogo incongruent trials (all *t* < −1.306, all *p* > 0.97).

## Data Availability

The data that support the findings of this study are available from the corresponding author upon reasonable request.
