# Supplementary material for: Distinct Alpha Connectivity Patterns During Response Inhibition in Alcohol Use Disorder
Source: Hum Brain Mapp. 2025 Sep 24;46(14):e70338. doi: 10.1002/hbm.70338 (PMC12459743; doi:10.1002/hbm.70338)
Supplement: Supplementary file 1 — Table S1: Pearson's correlations. Table S2: Number of standard drinks per serving size of different alcoholic beverages in liters (l). Table S3: Frequency table for AUD 1‐year criteria and AUD lifetime criteria for AUD and control participants. Figure S1: Behavioral results. The box plots illustrate the mean accuracy in percent (%) for the four experimental conditions. Accuracy in the Go condition was measured in correct responses. Accuracy for the Nogo condition was a measure of correct omission. Asterisks (*) indicate significant differences at p < 0.05. Error bars represent the 95% confidence interval. The performance of participants with alcohol use disorder (AUD) is presented in blue, while that of healthy controls is presented in green. Figure S2: Time‐frequency representation averaged over all channels for AUD (top panels) and control group (bottom panels) for Nogo congruent and Nogo incongruent trials in the time frame used for the nCREANN analysis (1 s after stimulus onset). No significant difference was found between Nogo congruent and Nogo incongruent in alpha and theta band power for the AUD (all t < 1.029, all p > 0.308) and the control groups (all t < 1.542, all p > 0.128). No significant difference was also present between the AUD and the Control group in alpha and theta frequency for Nogo congruent trials (All t < −1.420, all p > 0.79) and Nogo incongruent trials (all t < −1.306, all p > 0.97). [file HBM-46-e70338-s001.docx]

**Supplement to: Distinct Alpha Connectivity Patterns During Response Inhibition in Alcohol Use Disorder**

Filippo Ghin^#,1^, Nasibeh Talebi^1^, Ann-Kathrin Stock^1,*^, Christian Beste^1,*^

# Pearson`s Correlations between BDI and Education in years scores and behavioral performance.

**Table S1**: Pearson`s Correlations

|  |  | **Go**  **Congruent** | **Go Incongruent** | **Nogo**  **Congruent** | **Nogo**  **Incongruent** |
| --- | --- | --- | --- | --- | --- |
| **Controls** | **BDI** | *r*=-0.04, *p*=0.75 | *r*=-0.06, *p*=0.66 | *r*=0.01, *p*= 0.94 | *r*=-0.01, *p*= 0.96 |
|  | **Years of education** | *r*=0.01, *p*= 0.95 | *r*=0.38, *p*= 0.38 | *r*=0.1, *p*=0.40 | *r*=0.04, *p*=0.78 |
| **AUD** | **BDI** | *r*=-0.13, *p*= 0.34 | *r*=-0.12, *p*=0.35 | *r*=-0.06, *p*=0.68 | *r*=-0.11, *p*= 0.43 |
|  | **Years of education** | *r*=-0.17, *p*= 0.22 | *r*=-0.16, *p*= 0.23 | *r*=-0.15, *p*= 0.28 | *r*=-0.8, *p*= 0.58 |
| **All Participants** | **BDI** | *r*=-0.11, *p*= 0.24 | *r*=-0.09, *p*= 0.34 | *r*=0.06, *p*= 0.52 | *r*=0.02, *p*= 0.83 |
|  | **Years of education** | *r*=-0.08, *p*= 0.38 | *r*=0.01, *p*= 0.92 | *r*=0.12, *p*= 0.21 | *r*=0.07, *p*= 0.45 |

** Correlation is significant at the 0.01 level (2 tailed).

*Correlation is significant at the 0.05 level (2 tailed).

# Drinking frequency conversion table from alcoholic beverages in liters (l) to the number of standard drinks

**Table S2.** Number of standard drinks per serving size of different alcoholic beverages in liters (l).

| **Type of alcoholic beverage** | **Conversion of beverage quantity to standard drinks** | | |
| --- | --- | --- | --- |
| Beer | 0.3l = 1.5 drinks | 0.5 = 2.5 drinks | 1l = 5 drinks |
| Wine | 0.2l = 1.8 drinks | 0.7 = 6.5 drinks | 1.0l = 9 drinks |
| Liquor wine | 0.05l = 0.75 drinks | 0.1l =1.5 drinks | 0.2l = 3 drinks |
| Sparkling wine | 0.1l = 1 drink | 0.2l = 2 drinks | 0.75l = 8 drinks |
| Spirits & hard liquors | 0.02l = 0.75 drinks | 0.04l = 1.5 drinks | 0.7l = 25 drinks |
| Sweet liquors | 0.02 = 0.5 drinks | 0.04l = 2 drinks | 0.08l = 4 drinks |
| Cocktails | 0.02l = 0.75 drinks | 0.04l = 1.5 drinks | 0.08l = 3 drinks |

# Detailed frequency table of 1-year and lifetime AUD criteria in each group

**Table S3**: Frequency table for AUD 1-year criteria and AUD lifetime criteria for AUD and control participants.

| **1-year AUD criteria** | **AUD (n = 59)** | | **Controls (n = 64)** | |
| --- | --- | --- | --- | --- |
|  | ***N*** | ***Percentage*** | ***N*** | ***Percentage*** |
| 0 | 0 | 0 | 48 | 75% |
| 1 | 0 | 0 | 16 | 25% |
| 2 | 6 | 10.2% | 0 | 0 |
| 3 | 12 | 20.3% | 0 | 0 |
| 4 | 19 | 32.2% | 0 | 0 |
| 5 | 8 | 13.6% | 0 | 0 |
| 6 | 6 | 10.2% | 0 | 0 |
| 7 | 5 | 8.5% | 0 | 0 |
| 8 | 2 | 3.4% | 0 | 0 |
| 9 | 1 | 1.7% | 0 | 0 |
| 10 | 0 | 0 | 0 | 0 |
| **Lifetime AUD criteria** | ***N*** | ***Percentage*** | ***N*** | ***Percentage*** |
| 0 | 4 | 6.8% | 39 | 75% |
| 1 | 5 | 8.5% | 13 | 25% |
| 2 | 10 | 16.9% | 8 | 12.5% |
| 3 | 6 | 10.2% | 3 | 4.7% |
| 4 | 7 | 11.9% | 1 | 1.6% |
| 5 | 12 | 20.3% | 0 | 0 |
| 6 | 3 | 5.1% | 0 | 0 |
| 7 | 4 | 6.8% | 0 | 0 |
| 8 | 5 | 8.5% | 0 | 0 |
| 9 | 1 | 1.7% | 0 | 0 |
| 10 | 2 | 3.4% | 0 | 0 |
| *Note: All of the participants included in the control group (i.e., AUD 1-year criteria ≤1) who reported lifetime AUD criteria ≥ 2 were drinking within normal range for the past 3 months prior to the experiment (i.e., drinking frequency always ≥1). | | | | |

# Behavioral results

Figure 2 illustrates behavioral performance (accuracy) in all conditions of the Simon Nogo task, which were previously published in Ghin et al. (2022). Behavioral data were analyzed using repeated-measures ANOVAs with condition (Go vs. Nogo) and congruency (congruent vs. incongruent) as within-subject factors and group (AUD vs. controls) as between-subject factor. When necessary, a Greenhouse-Geisser correction was applied to account for the possible lack of sphericity. All variables were tested for normal distribution using Kolmogorov-Smirnov tests. When the assumption of normal distribution was violated, additional non-parametric Wilcoxon signed-rank tests were used. Bonferroni correction for multiple comparisons was applied when necessary. Descriptive statistics are reported using the mean value and the standard error of the mean (SEM).

The repeated-measures ANOVA for accuracy showed that there were significant main effects of condition (*F*_(1,121)_ = 24.856, *p* < 0.001, *η*^2^*_p_* = 0.170), congruency (*F*_(1,121)_ = 10.533, *p* = 0.002, *η*^2^*_p_* = 0.080) and group (*F*_(1,121)_ = 8.120, *p* = 0.005, *η*^2^*_p_* = 0.063). Specifically, there was a higher accuracy for Go trials (97.33% ± 0.17) compared to Nogo trials (95.44% ± 0.40), and higher accuracy in incongruent (96.61% ± 0.24) than in congruent trials (96.16% ± 0.26). Furthermore, the AUD the group showed an overall higher accuracy performance than the control group (AUD = 97.06% ± 0.35; control = 95.7% ± 0.33). To explore the typical task effects, interactions were also explored. A significant condition x congruency interaction was found (*F*_(1,121)_ = 42.296, *p* < 0.001, *η*^2^*_p_* = 0.259). Bonferroni-corrected post-hoc comparisons revealed a significantly (*t* = 3.629, *p* = 0.002) higher accuracy for Go congruent trials (97.65% ± 0.16) than Go incongruent trials (97.01% ± 0.2). Furthermore, as expected, an inverse pattern was found for Nogo trials (*t* = −6.087, *p* = 0.00) with higher accuracy for Nogo incongruent (96.15% ± 0.38) than Nogo congruent (94.60% ± 0.48) trials. Additional Wilcoxon signed-rank tests confirmed these findings for Go (*z* = −3.049, *p* = 0.004) and Nogo trials (*z* = −5.234, *p* = 0.002). A significant three way interaction of condition x congruency x group was found (*F*_(1,121)_ = 4.414, *p* = 0.038, *η*^2^*_p_* = 0.035). In order to investigate this interaction, additional post-hoc repeated-measures ANOVAs with congruency as within-subject factor and group as between-subject factor were conducted separately for the Go and Nogo condition. In the Go condition, there was a significant main effect of congruency (*F*_(1,121)_ = 12.866, *p* < 0.001, *η²ₚ* = 0.096), with performance being higher in the congruent trials (97.64% ± 0.16) compared to incongruent trials (97.02% ± 0.21). However, there was no significant main effect of group (*F*_(1,121)_ = 0.052, *p* = 0.820, *η²ₚ* < 0.001), nor was there a significant interaction between congruency and group (*F*_(1,121)_ = 0.794, *p* = 0.375, *η²ₚ* = 0.007). For the Nogo condition, a significant main effect of congruency (*F*_(1,121)_ = 37.016, *p* < 0.001, *η*^2^*_p_* = 0.234) with higher accuracy in incongruent (96.20% ± 0.37) than congruent trials (94.67% ± 0.46), and a significant main effect of group (*F*_(1,121)_ = 12.323, *p* < 0.001, *η*^2^*_p_* = 0.092) with higher accuracy for the AUD group (96.84% ± 0.58) than for the control group (94.03% ± 0.55) was found. Most interestingly, a congruency x group interaction was found for Nogo trials (*F*_(1,121)_ = 4.628, *p* = 0.033, *η*^2^*_p_* = 0.037). Bonferroni-corrected post-hoc comparisons revealed that compared to the control group, the AUD group had significantly higher accuracy in both congruent (*t* = −3.724, *p* = 0.002; AUD= 96.34% ± 0.41, Control=93%± 0.80) and incongruent trials (*t* = −3.109, *p* = 0.004; AUD=97.33% ± 0.36, Control=95.07% ± 0.63). These results were confirmed by non-parametric statistical analyses (all *z* ≥ |2.647|, all *p* ≤ 0.016).

**
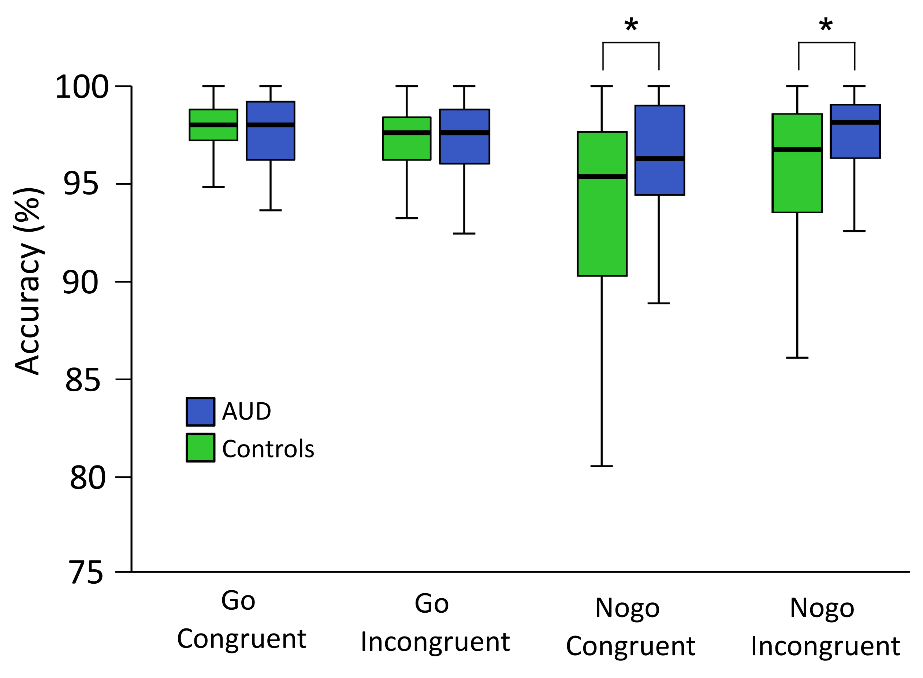
**

**Figure S1**: Behavioral results. The box plots illustrate the mean accuracy in percent (%) for the four experimental conditions. Accuracy in the Go condition was measured in correct responses. Accuracy for the Nogo condition was a measure of correct omission. Asterisks (*) indicate significant differences at p<0.05. Error bars represent the 95% confidence interval. The performance of participants with alcohol use disorder (AUD) is presented in blue, while that of healthy controls is presented in green.

We also investigated whether AUD lifetime criteria might play a role in the behavioral result. To do this, a two-way mixed effect ANCOVA with condition (Go vs. Nogo) and congruency (congruent vs. incongruent) as within-subject factors, group (AUD vs. control group) as the between-subjects factor and AUD lifetime criteria as single covariate was used.. Results showed that there was a main effect of condition (*F*_(1,121)_ = 15.258, *p* < 0.001, *η*^2^*_p_* = 0.113) with higher accuracy for Go trials (97.34%±0.16) compared to Nogo, trials (95.43%±=0.40) and group with higher accuracy in the AUD group (97.16%±0.42)compared to the Control group(95.61%±0.40). No significant main effect of congruency and AUD lifetime criteria was found (*F*≤ 2.143, *p*≥0.146). A significant Condition x Congruency interaction (*F*_(1,121)_ =15.240 *p* < 0.001, *η*^2^*_p_* = 0.113) and Condition x Group (*F*_(1,121)_ =4.417 *p* = 0.038, *η*^2^*_p_* = 0.036) were found. Bonferroni-corrected post-hoc comparisons revealed a significantly (*t* = 3.629, *p* = 0.002) higher accuracy for Go congruent trials (97.65% ± 0.16) than Go incongruent trials (97.01% ± 0.2). Furthermore, as expected, an inverse pattern was found for Nogo trials (*t* = −6.087, *p* = 0.00) with higher accuracy for Nogo incongruent (96.15% ± 0.38) than Nogo congruent (94.60% ± 0.48) trials. Importantly, Bonferroni post-hoc corrected comparison showed that the AUD group showed higher accuracy in Nogo condition compared to the Control group (*t*=3.601, *p*<=0.002; AUD=96.83% ± 0.35, Controls =94.03% ± 0.7). However, no significant difference was present for Go condition between AUD and Control group (*t*= 0.228, *p*=0.41). No other significant interaction was shown.

Overall, the results from the two-way mixed ANCOVA showed that group differences in automatic vs. controlled processes in response inhibition might be modulated by the AUD lifetime criteria as evidenced by the changes in the in the interaction of group × condition × congruency in the main analysis. However, it is critical to note that there was a rather large overlap between those participants who presented with at least two AUD lifetime and AUD last year criteria. Also, as a consequence of this, it is worth noting that the better inhibitory response performance in AUD, compared to the Control group, still holds when investigating lifetime AUD

1. **Time-Frequency Data**


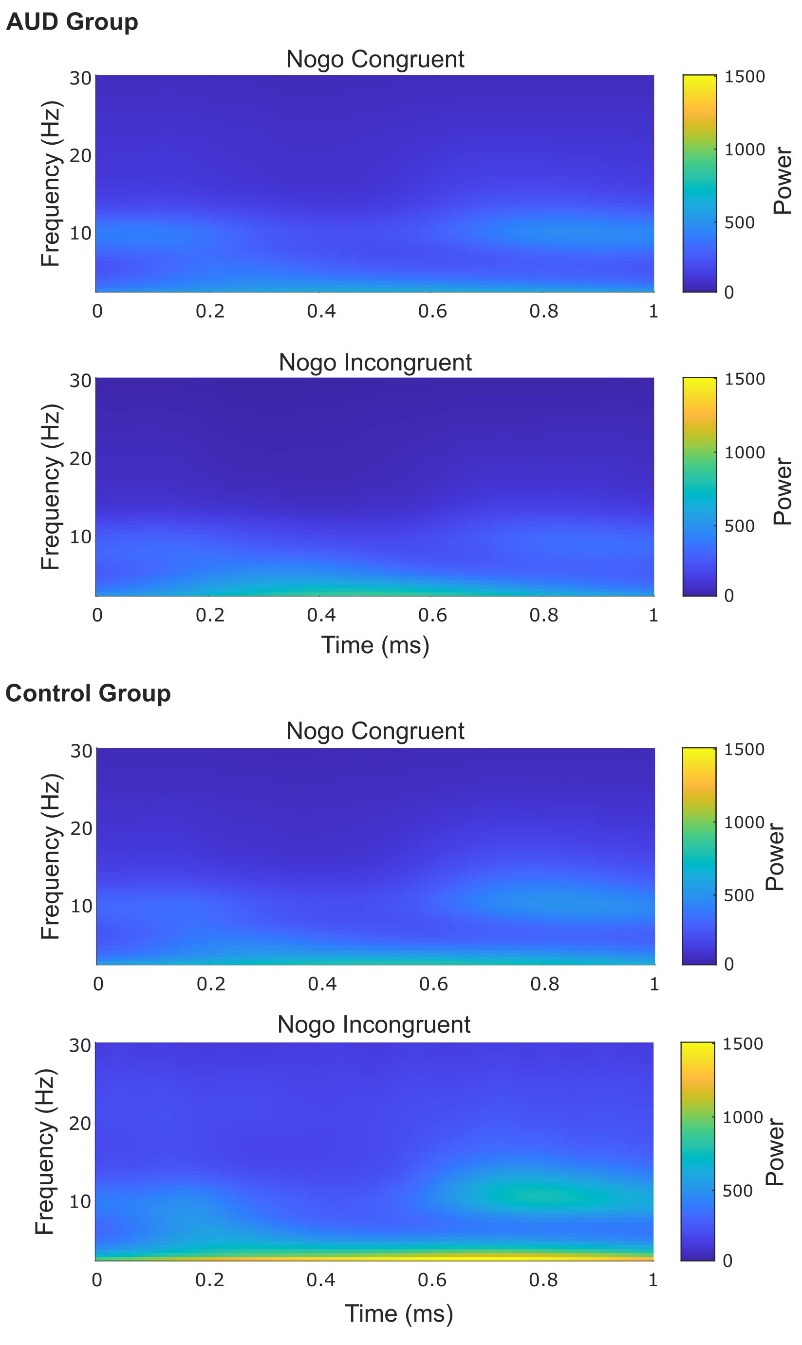


Figure 2S: Time-frequency representation averaged over all channels for AUD (top panels) and control group (bottom panels) for Nogo congruent and Nogo incongruent trials in the time frame used for the nCREANN analysis (1 sec after stimulus onset). No significant difference was found between Nogo congruent and Nogo incongruent in alpha and theta band power for the AUD (all *t*< 1.029, all *p*>0.308) and the control groups (all *t*<, all *p*>0.128). No significant difference was also present between the AUD and the Control group in alpha and theta frequency for Nogo congruent trials (All *t*<-1.420, all *p*>0.79) and Nogo incongruent trials (all *t*<-1.306, all *p*>0.97).
